# Normal vision can compensate for the loss of the circadian clock

**DOI:** 10.1098/rspb.2015.1846

**Published:** 2015-09-22

**Authors:** Matthias Schlichting, Pamela Menegazzi, Charlotte Helfrich-Förster

**Affiliations:** Neurobiology and Genetics, Theodor Boveri Institute, Biocenter, University of Würzburg, Würzburg, Germany

**Keywords:** circadian rhythms, period, timeless

## Abstract

Circadian clocks are thought to be essential for timing the daily activity of animals, and consequently increase fitness. This view was recently challenged for clock-less fruit flies and mice that exhibited astonishingly normal activity rhythms under outdoor conditions. Compensatory mechanisms appear to enable even clock mutants to live a normal life in nature. Here, we show that gradual daily increases/decreases of light in the laboratory suffice to provoke normally timed sharp morning (M) and evening (E) activity peaks in clock-less flies. We also show that the compound eyes, but not Cryptochrome (CRY), mediate the precise timing of M and E peaks under natural-like conditions, as CRY-less flies do and eyeless flies do not show these sharp peaks independently of a functional clock. Nevertheless, the circadian clock appears critical for anticipating dusk, as well as for inhibiting sharp activity peaks during midnight. Clock-less flies only increase E activity after dusk and not before the beginning of dusk, and respond strongly to twilight exposure in the middle of the night. Furthermore, the circadian clock responds to natural-like light cycles, by slightly broadening Timeless (TIM) abundance in the clock neurons, and this effect is mediated by CRY.

## 1. Background

The daily pattern of animal behaviour is thought to be of critical importance for fitness. It is generally assumed that the circadian clock times activity to the optimal time of day, and that possessing a circadian clock is important for survival and reproductive fitness (e.g. [1–3]). This view was recently challenged in mice and fruit flies, because clock-less mutants showed almost wild-type (WT) activity patterns when exposed to natural-like conditions [4–7]. In particular, WT mice and mutants of the *period2* gene (*Per2^Brdm1^*), kept in a semi-natural outdoor environment over 2 years, displayed negligible differences in activity patterns while exhibiting considerable and similar seasonal adaptations [4]. Furthermore, the clock mutation had no persistent negative effects on fitness. In fruit flies, locomotor activity of WT and different clock-less mutants was recorded in the traditional glass tubes placed outdoors in an area sheltered from rain and direct sunlight [5–7]. All genotypes showed typical morning (M) and evening (E) activity, and virtually no differences in activity patterns were observed between clock-less mutants and WT flies.

To determine the temporal cues that enable clock-less flies to time locomotor activity in a WT manner in nature, we recorded locomotor activity of the wild-type strain CantonS (WT_CantonS_) and of previously studied clock mutants—*per^01^* and *tim^01^*—in the laboratory, where we could precisely define the cyclic environmental conditions. We kept temperature and day length constant, and only varied the daily light profile: all flies were first exposed to usual laboratory light–dark (LD) cycles, with the light being switched on/off suddenly, then to LD cycles with simulated twilight (LDR1, R = ramp [8,9]), and finally to LD cycles closely mimicking the light profile occurring in nature (LDR2 [10,11]). We found that the natural-like light profile was sufficient to provoke almost WT-like activity patterns in the mutants.

Next we aimed to unravel the light-input pathways that are responsible for WT-like activity pattern of the mutants. *Drosophila melanogaster* has several photoreceptors: the compound eyes, the ocelli, the extraretinal eyelets and Cryptochrome (CRY; reviewed in [12]). Among these, CRY and the compound eyes have the greatest impact on locomotor activity rhythms. CRY is expressed in the majority of *Drosophila*'s lateral and a subset of its dorsal clock neurons [13,14]. Upon light activation, CRY interacts directly with the molecular feedback loop that generates circadian oscillations by provoking degradation of the clock protein Timeless (TIM) [15,16]. Consequently, the molecular clock is set to a new phase [17]. The compound eyes only have moderate effects on rhythm phase [18], but they clearly interact with the clock and contribute to proper entrainment to LD cycles [19]. Furthermore, the eyes are necessary to adapt fly activity to long days [20], to nocturnal dim light [21,22] and to twilight [23].

To investigate whether CRY or the compound eyes are necessary for a WT-like behaviour under natural-like light cycles, we recorded mutants without CRY (*cry^01^* mutants [24]), without eyes (*cli^eya^* mutants [22,25]), and mutants that lack the clock and additionally either CRY or the eyes under the above-mentioned light schedules. Furthermore, we measured TIM oscillations in the lateral clock neurons of WT flies and *cry^01^* mutants to reveal the effect of natural-like light cycles on the molecular clock. We found that CRY is responsible for slight effects of natural-like light cycles on the molecular clock, but that the compound eyes are responsible for fly WT-like activity patterns.

## 2. Material and methods

### (a) Fly strains and rearing

To investigate the impact of a functional clock on locomotor activity, we studied *per^01^* and *tim^01^* flies, null mutants for the core clock genes *per* and *tim* [26–28]. WT_CantonS_ served as general control whereas the WT_Lindelbach_ [22] served as additional control for assessing TIM cycling. *cli^eya^* mutants, which lack compound eyes but have normal ocelli as well as normal clock neurons and extraretinal Hofbauer-Buchner eyelets [22,25], were used to investigate the role of compound eyes in timing activity to the appropriate time of day. *cry^01^* mutants [24] and *cli^eya^*;*cry^b^* double mutants [20] were used to test the role of CRY in this process. Mutants were backcrossed to WT_CantonS_ for five generations to ensure the same genetic background. Double mutants, *per^01^;cli^eya^* and *per^01^;;cry^01^*, were used to investigate the importance of both intact vision and a functional clock on timing of behaviour. All flies were raised on *Drosophila* medium (0.8% agar, 2.2% sugarbeet syrup, 8.0% malt extract, 1.8% yeast, 1.0% soy flour, 8.0% corn flour and 0.3% hydroxybenzoic acid) at 25°C in LD 12 : 12.

### (b) Locomotor activity recordings and data analysis

Fly locomotion was measured in a homemade system described first by Helfrich-Förster [29] and refined by Rieger *et al*. [8]. Two-to five-day-old male flies were singly transferred into photometer cuvettes with water and food supply on one end and an infrared light-beam recording the number of infrared light-beam interruptions caused by the fly in 1 min intervals on the other end. All experiments were performed in a climate-controlled chamber at 20°C. Illumination was provided by tunable ‘white’ LEDs (Lumitronix LED-Technik GmbH, Jungingen, Germany). In addition, neutral density filters (Lee Filters Worldwide, Hampshire, UK) were used for fine adjustment of light intensity. We simulated three different light conditions, each consisting of 12 h light and 12 h darkness, and a maximal light intensity of 100 lux. In LD, we simulated a rectangular LD cycle; in LDR1 the light intensity increased/decreased logarithmically in 1 min steps within 1.5 h in the morning and evening to simulate dawn and dusk [8]; and in LDR2 the light intensity increased/decreased within 4.5 h to simulate the course of the sun within 1 day. The dark period remained the same (12 h) for all conditions. Each light condition was given for 7 days with LD being present from day 1 to 7, LDR1 from day 8 to 14 and LDR2 from day 15 to 21. In each experiment, 32 flies per genotype were recorded, but only flies surviving until day 21 were analysed. To further test the contribution of masking in the response to simulated twilight, we entrained flies in LD 12 : 12 for 6 days. On day 6, we additionally applied a light pulse in the night, with light intensity rising to 100 lux between ZT15.5 and ZT17, and decaying between ZT19 and ZT20.5. For testing the entrainability of *cli^eya^*;*cry^b^* double mutants, we entrained them first for 8 days to LD12 : 12 and then phase-delayed the LD by 6 h to see whether the mutants can follow the phase shift (see the electronic supplementary material, figure S1).

Raw data were plotted as actograms using ActogramJ [30]. Behavioural analysis was performed as described by Schlichting & Helfrich-Förster [31]. Statistical analysis was performed using Systat11. After testing for normal distribution by a Kolmogorov–Smirnov test, data were compared using a two- or three-way ANOVA. In the case of data not normally distributed, *p*-values were adjusted by multiplication by 5 [32].

### (c) Fluorescent immunohistochemistry

To analyse the molecular cycling of TIM in the brain, 1–4-day-old male WT_CantonS_, WT_Lindelbach_ and *cry^01^* flies were entrained for 5 days either in LD or LDR2 with a maximal light intensity of 100 lux and sampled every 1–2 h. Whole flies were fixed in 4% paraformaldehyde (PFA) in phosphate buffer (PB) containing 0.1% Triton X-100 (PBT, pH = 7.4) for 2.5 h at room temperature. After washing the flies four times for 15 min in PB, the brains were dissected in PB and afterwards transferred into blocking solution comprising 5% normal goat serum (NGS) in PBT overnight at 4°C. On the following day, the brains were transferred into the first antibody solution containing rat anti-TIM (dilution 1 : 1000; provided by Isaac Edery [33]) and mouse anti-PDF (dilution 1 : 2000; Developmental Studies Hybridoma Bank, Iowa), 5% NGS and 0.02% NaN_3_ in PBT. After overnight incubation at 4°C, the brains were washed five times in PBT for 10 min. In the next step, the secondary antibody solution, consisting of Alexa Fluor 555 (goat anti-rat) and Alexa Fluor 635 (goat anti-mouse), each in a dilution of 1 : 200 in PBT containing 5% NGS, was applied for 3 h at room temperature. After washing five times for 10 min each in PBT, brains were embedded in Vectashield mounting medium (Vector Laboratories, Burlingame, CA, USA) with the anterior surfaces facing up on the slide.

### (d) Microscopy and image analysis

Brains were analysed using laser scanning confocal microscopy (Leica TCS SPE; Leica, Wetzlar, Germany). To excite the fluorophores of the secondary antibodies, we used two different laser diodes (532 and 635 nm) and obtained confocal stacks of 2 µm thickness. To quantify and compare the intensity of TIM staining, laser settings were kept constant for all samples. Staining intensity was analysed in all clock neuron clusters except for the DN_3_ using the ImageJ distribution package Fiji (http://fiji.sc/Fiji). For quantification, we determined the brightness of single clock neurons using a 9-pixel area and subtracted three different background intensities to compensate for unspecific staining as described by Menegazzi *et al*. [34]. For each time-point, one hemisphere of at least five different brains was analysed. TIM cycling was normalized to 1 and plotted using Qtiplot v. 0.9.8.9 (Ion Vasilief, Craiova, Romania). To show general tendencies in TIM staining intensity cycling, a polynomial fit of the fourth order considering the value's standard error of the mean was applied (internal function of Qtiplot). A three-way ANOVA was used to calculate the dependency of TIM increase/decrease on time (ZT), light regime (LD/LDR2) and clock neuron group.

## 3. Results

### (a) M and E peak timing under simulated twilight is independent of a functional clock, but the clock is necessary for anticipation of dusk

**Figure 1.**
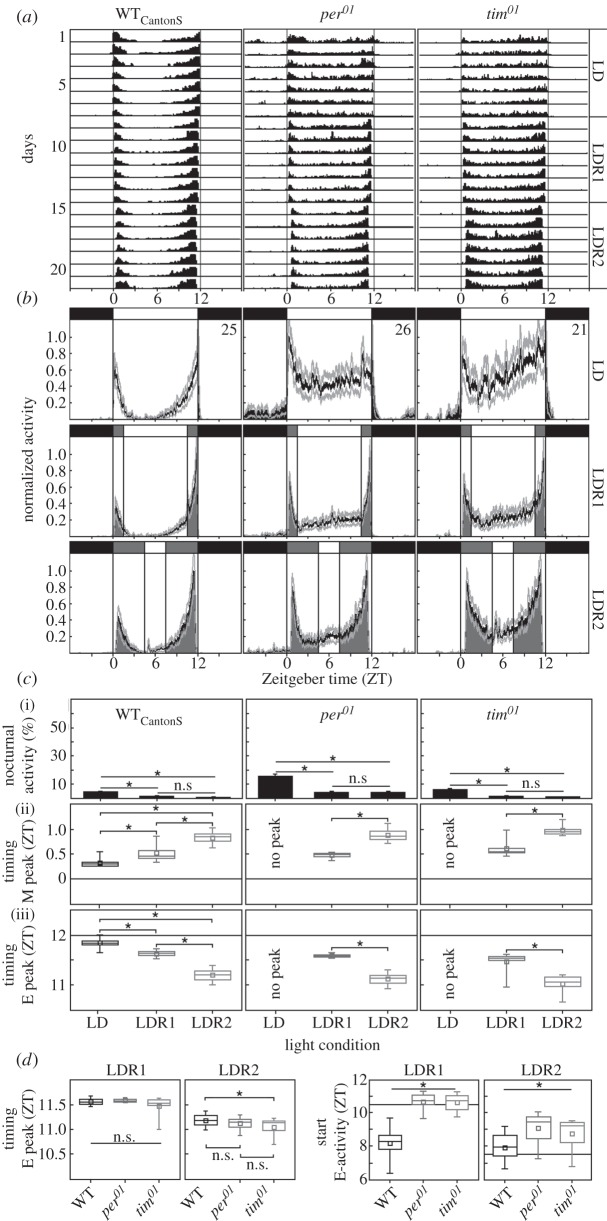
Rhythmic activity of wild-type flies (WT_CantonS_) and of the clock mutants *per^01^* and *tim^01^* in LD cycle (with lights on and off), LDR1 (LD with 1.5 h twilight simulation) and LDR2 (LD with 4.5 h increasing/decreasing light). For each strain, (*a*) average actograms, (*b*) average activity profiles, and (*c*) nocturnal activity and timing of morning (M) and evening (E) activity peaks were calculated. (*a*) The mean activity of 25 flies is indicated in black without error bars. The grey vertical lines indicate Zeitgeber time (ZT) 0 (i.e. beginning of day) and ZT 12 (i.e. beginning of night). (*b*) The average activity profiles indicate the average activity of all flies (black curve) ±s.e.m. (light grey) under the relevant light condition (LD, LDR1 and LDR2), which is given on top of each diagram (black, complete darkness; dark grey, time of increasing or decreasing light intensity; white, time of maximal light intensity (100 lux)). The WT strain shows bimodal activity patterns with M and E activity bouts under all three light conditions. (*c*) Upon LDR1 and LDR2, (i) nocturnal activity decreases, and (ii) M and (iii) E peaks delay/advance, respectively. Nocturnal activity is expressed as a percentage of whole daily activity (±s.e.m.), timing of M and E peak in ZT. Clock mutants lack bimodal activity patterns under LD, but develop them under LDR1 and LDR2 (*a*,*b*). Nocturnal activity is higher than in WT flies under LD, but is reduced under LDR1 and LDR2 in a WT-like manner (*c*). Timing of M and E peaks under LDR1 and LDR2 is also WT-like in the mutants (*c*). Consequently, the activity pattern of *per^01^* and *tim^01^* mutants is virtually indistinguishable from that of WT flies in LDR2 (*b*). Only activity during the siesta is higher and the onset of E activity is later (*d*) in the mutants. For direct comparison, the timing of the E peaks and the onset of E activity are plotted in a comparative manner in (*d*). Whereas E peak timing appears similar in all fly strains, the start of E activity is clearly later in the mutants than in the WT flies. Asterisks indicate significant differences between values.

WT flies showed the typical bimodal activity pattern, with M and E activity bouts, and a pronounced siesta between them under all tested conditions (LD, LDR1 and LDR2; figure 1). In LD, M activity peaked shortly after lights on, E activity peaked shortly before lights off and the flies were strongly diurnal as maximally 11.7% activity took place at night. In contrast to previous studies [35], WT flies completely lacked M anticipatory activity. This lack of M anticipation can be explained by our particular recording system and by the rather low temperature of 20°C (see the electronic supplementary material). Dawn and dusk simulation even augmented fly diurnality (figure 1*c*), since the M peak significantly delayed and the E peak significantly advanced in LDR1, and even further in LDR2 (figure 1*d*,*e*; M peak: *F*_2,51_ = 62.573, *p* < 0.001; E peak: *F*_2,52_ = 84.966, *p* < 0.001).

As reported previously [36–39], *per^01^* and *tim^01^* clock mutants behaved very differently from WT flies under conventional LD cycles: they showed no clear M and E activity bouts, lacked the siesta and were slightly more active during the night (figure 1). When light intensity was gradually increased/decreased (LDR1 and LDR2), the locomotor activity of *per^01^* and *tim^01^* mutants became more similar to the one of WT flies: WT-like M and E peaks appeared (figure 1*b*) and nocturnal activity decreased (figure 1*c*). Mutant flies delayed the M peak and advanced the E peak in LDR2 compared to LDR1, as WT flies did (figure 1*c*). Two-way ANOVAs revealed that the timing of the E peak and nocturnal activity depended significantly on the light condition (LDR1/LDR2) as well as on the genotype (electronic supplementary material, table S1). Pairwise *post hoc* comparisons showed that all values marked by asterisks in figure 1 are significantly different. Under LDR2, the activity pattern of the mutants was virtually indistinguishable from that of WT flies; only the activity during the siesta remained higher in the mutants (figure 1*b*), and the increase in E activity was later in the mutants than in WT flies (figure 1*d*).

Taken together, our findings show that the timing of M and E peaks, as well as the shift of activity out of the night into daytime upon simulation of natural-like light conditions, is independent of a functional clock. The two clock mutants are able to precisely track changes in light intensity with their activity. Consequently, the activity patterns of *per^01^* and *tim^01^* mutants look surprisingly similar to those of WT flies. When calculating the light intensity at which M and E peaks occurred, we found that they always took place between 1 and 10 lux, independently of the presence or the absence of the clock. By contrast, the anticipation of dusk clearly depended on the clock (figure 1*d*). The clock-less mutants started to increase E activity just at the onset of dusk simulation, whereas WT flies started their E activity already before dusk.

### (b) The compound eyes are necessary to time M and E peaks in a wild-type manner

**Figure 2.**
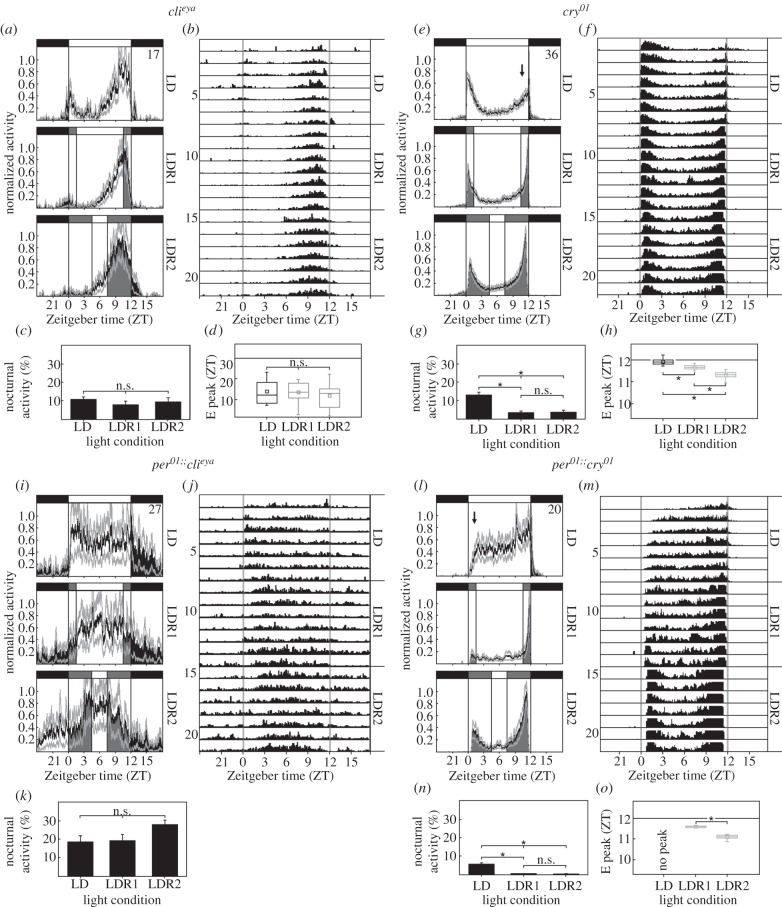
Rhythmic activity of mutants (*a*–*h*) with impaired photoreception (*cli^eya^* and *cry^01^* mutants) and (*i*–*o*) with impaired photoreception plus circadian clock (*per^01^;cli^eya^* and *per^01^;;cry^01^* double mutants) in LD, LDR1 and LDR2. Average actograms, average activity profiles as well as nocturnal activity and the timing of the E peaks are shown (labelling as in figure 1). Only for *per^01^;cli^eya^* double mutants could E peak times not be calculated, because E peaks were simply absent (*i*,*j*). *cli^eya^* mutants lack the sharp M and E peaks but still show M and E activity bouts, although the M bout is small under LDR1 and LDR2 (*a*,*b*). *cli^eya^* mutants neither reduce nocturnal activity (*c*) nor advance their E activity maxima (*d*) in response to LDR1 and LDR2. *cry^01^* mutants behave in principal WT-like (*e*–*h*). Only their E activity rises slower under LD (arrow). *per^01^;cli^eya^* double mutants still respond to the light regimes and even modify their activity pattern in response to LDR1 and LDR2, but they do not show M and E activity bouts nor sharp M and E peaks under any condition (*i*, *j*). Nocturnal activity levels also do not change in response to LDR1 and LDR2 (*k*). *per^01^;;cry^01^* double mutants behave in principle like *per^01^* single mutants, with the exception that their activity after lights on is lower (arrow) under LD and the M peak rather small under LDR1 (*l*,*m*).

We subsequently wanted to elucidate the mechanisms by which flies precisely time their activity peaks under LDR even in the absence of a functional clock. We therefore tested *cli^eya^* and *cry^01^* mutants under LD, LDR1 and LDR2 conditions (figure 2*a–h*). We found that both mutants still entrained to all applied light conditions, but that their activity patterns differed substantially from each other. *cry^01^* mutants behaved essentially indistinguishably from WT flies, whereas *cli^eya^* mutants exhibited rounded M and E activity bouts but no sharp M and E peaks. Similar activity patterns of flies without functional eyes have previously been reported [20,36,40]. Two-way ANOVAs revealed significant effects of light condition and genotype on E peak timing as well as on nocturnal activity (electronic supplementary material, table S1). Most importantly, *post hoc* analysis showed that WT flies and eyeless mutants had significantly different E activity timing and nocturnal activity levels, whereas *cry^01^* mutants did not differ from WT flies in either respect (peak timing: *p* = 0.708; nocturnal activity *p* = 0.064).

In summary, *cli^eya^* mutants neither reduced nocturnal activity when exposed to LDR1 and LDR2 conditions (figure 2*c*) nor significantly altered the timing of E activity bouts upon these conditions (figure 2*d*). We could not reliably calculate the phase of the M activity bout in *cli^eya^* mutants, because M activity was drastically reduced under LDR1 and even more so under LDR2 (figure 2*a*). This shows that *cli^eya^* mutants can still detect the different light conditions but that the compound eyes are absolutely essential for the presence of sharp M and E peaks, and their precise timing to gradually increasing/decreasing light. CRY, on the other hand, appears unnecessary for this timing, but it is clearly involved in the timing of the rounded activity bouts of *cli^eya^* mutants. *cli^eya^*;*cry^b^* double mutants that lack functional CRY in addition to the compound eyes are barely able to entrain to LD cycles [20]. Here we show that *cli^eya^*;*cry^b^* double mutants do not entrain to LDR1 cycles and that they cannot re-entrain to a 6 h delay of the LD cycle (electronic supplementary material, figure S1). In summary, this shows that either the compound eyes or CRY alone are sufficient for entrainment to LD cycles and that normally both cooperate in determining the timing of M and E activity. The compound eyes are necessary for the appearance and timing of sharp M and E peaks, whereas CRY is essential for entraining the rounded M and E activity bouts that can be regarded as endogenous property of the circadian clock [41].

### (c) Functional eyes are also necessary for wild-type-like activity patterns in clock mutants

To test whether losing the compound eyes but not CRY also leads to a loss of M and E activity peaks in clock-less flies, we recorded *per^01^;cli^eya^* and *per^01^;;cry^01^* double mutants under LD, LDR1 and LDR2 conditions (figure 2*i*–*o*). Indeed, the activity pattern of *per^01^;;cry^01^* double mutants was very similar to that of *per^01^* single mutants (compare figure 2*l*–*o* with figure 1). By contrast, *per^01^;cli^eya^* double mutants lacked M and E peaks (figure 2*i*–*k*), showing that the occurrence of these peaks depends on functional compound eyes but not on CRY. We conclude that the normal timing of M and E peaks in *per^01^* mutants is solely caused by the ability of the compound eyes to measure and integrate the regularly changing light intensity and to provoke almost normal activity patterns under LDR2 even in the absence of a circadian clock. In other words, the absence of the eyes has a huge effect on *per^01^* mutants, rendering their activity pattern irregular due to a complete lack of M and E activity bouts and sharp peaks. By contrast, WT flies can largely compensate for the loss of eyes. The only effect is that they lack the sharp M and E peaks (see above).

### (d) The clock is needed for a normal siesta and for suppressing the response to twilight during the night

Although timing of M and E peaks in WT flies and clock mutants under LDR is very similar, some differences in the activity pattern of the two strains are evident. The typical WT-like siesta seemed less pronounced in the mutants (figure 1*b*) and, at least under LD, clock mutants were slightly more active during the night. This suggests that a functional clock may suppress activity during midday and midnight. The suppression of activity by the clock during midday is well known under rectangular LD conditions in the laboratory (e.g. [36]). Here we show that the clock also suppresses midday activity under simulated twilight in the laboratory. To test the hypothesis that a functional clock also inhibits nocturnal activity, we simulated 1.5 h dawn followed (2 h later) by 1.5 h dusk in the middle of the night while recording fly activity. If our hypothesis holds true, then it should be more likely to induce a response to twilight in mutant than in WT flies.

We found that *per^01^* and *tim^01^* mutants responded to ‘midnight twilight’ exactly as they did to morning/evening twilight (electronic supplementary material, figure S2). The same was true for clock-less flies that lacked, in addition, CRY (*per^01^*;;*cry^01^* mutants)*.* Flies with a functional clock like WT and *cry^01^* also increased activity in response to light, but lacked the sharp activity peaks during midnight twilight, whereas eyeless flies did not respond at all to nocturnal light. Together this shows that twilight-driven behaviour at midnight is mediated by the compound eyes but suppressed by a functional clock.

### (e) LDR2 alters TIM cycling in clock neurons in a CRY-dependent way

**Figure 3.**
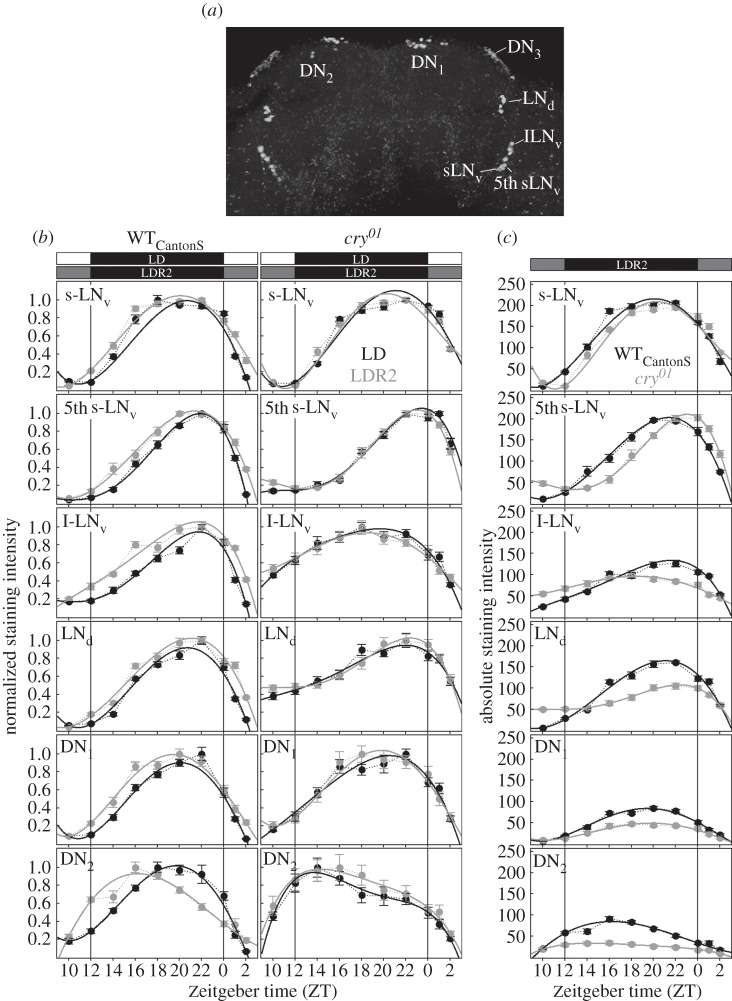
TIM cycling in the lateral clock neurons under LD and LDR2 in WT flies and *cry^01^* mutants. The position of the lateral (s-LN_v_, l-LN_v_, 5th s-LN_v_ and LN_d_) and dorsal (DN_1_, DN_2_ and DN_3_) clock neurons in the brain of the fly is indicated in (*a*), TIM cycling in these neurons in (*b*). Black circles (±s.e.m.) connected by thin broken black lines represent the measured staining intensity in LD, whereas grey circles (±s.e.m.) connected by thin grey dashed lines represent the staining intensity in LDR2. Polynomial fits of the cycling in LD and LDR2 are added in thick black and grey lines, respectively. The polynomial fits were characterized by *R*^2^ ≥ 0.98 indicating that they nicely match the original cycling. In WT flies, TIM accumulates earlier and stays stable for longer time in LDR2 when compared with LD in all neurons. In the DN_2_, the TIM peak is additionally advanced under LDR2. In *cry^01^* mutants, the LDR2 effects are absent. The direct comparison of TIM cycling in WT flies and *cry^01^* mutants under LDR2 (WT_CantonS_ in black and *cry^01^* in grey lines) (*c*) shows that TIM cycling is delayed in the 5th s-LN_v_ and flattened in the l-LN_v_, the LN_d_, the DN_2_ and to a minor degree in the DN_1_.

As the clock seems to contribute to the activity pattern of flies, we investigated next how LDR2 affects the molecular clock in the clock neurons (s-LN_v_, 5th s-LN_v_, l-LN_v_, LN_d_, DN_1_ and DN_2_; figure 3*a*). We assessed TIM cycling by immunocytochemical means in WT flies and *cry^01^* mutants under LD and LDR2.

As reported previously for WT flies under LD [16,26,42,43], we found TIM to rise after lights off, to reach its maximum between ZT18 and ZT21, and then to decrease again (figure 3*b*). After lights on, TIM disappeared and started to rise again after lights off (ZT12). More natural-like light conditions (LDR2) significantly affected TIM cycling in WT flies. Here, we tested two different WT strains, WT_CantonS_ (figure 3*b*) and WT_Lindelbach_ (electronic supplementary material, figure S3), that gave virtually the same results. A three-way ANOVA revealed that TIM staining during its increase (between ZT10 and ZT16 in WT_CantonS_ and between ZT12 and ZT18 in WT_Lindelbach_), as well as during its decrease (between ZT22 and ZT2 in both strains), depended (i) on the light condition (LD/LDR2), (ii) on time and (iii) on the clock neuron group (electronic supplementary material, table S2). Furthermore, there was a significant interaction between time of day and light condition on TIM, meaning that the slopes of TIM increase and decrease were different between LD and LDR2: TIM accumulated earlier and stayed stable for a longer time under LDR2 than under LD (figure 3*b*; electronic supplementary material, figure S3 and table S2). ANOVA also revealed a significant interaction between light condition and clock neuron groups, meaning that LDR2 influenced the different clock neurons differently: figure 3 and electronic supplementary material, figure S3 show that LDR2 broadened the TIM slopes most prominently in the 5th s-LN_v_ and the l-LN_v_. In the DN_2_, LDR2 additionally advanced the TIM peak (figure 3*b*; electronic supplementary material, figure S3).

*cry^01^* mutants did not show any significant difference in TIM cycling between LD and LDR2 (figure 3*b*; electronic supplementary material, table S2), indicating that CRY is necessary for mediating the observed effects of twilight on the clock. Nevertheless, the absence of CRY influenced the general TIM cycling profile in certain neurons. Consistent with earlier studies [19,21,44,45], the shape of TIM cycling was flattened in the l-LN_v_, LN_d_ and DN_2_ cells of *cry^01^* mutants, but also to some extent in the DN_1_ cells (figure 3*c*). Furthermore, TIM rose later and reached its peak significantly later in the 5th s-LN_v_ cell (E neuron) of *cry^01^* mutants as compared with WT flies (figure 3*c*)*.* The same tendency was visible in the LN_d_, though this was more difficult to judge because of the flattened shape of the curve.

## 4. Discussion

In nature, many environmental factors oscillate during the 24 h day, among which irradiance, temperature and humidity are most important. Animals sense these regular fluctuations and respond immediately in an adequate way. For example, a diurnal animal whose visual system cannot tune to darkness will stop moving after night onset. In addition, the circadian clock will prepare the animal for the coming night, allowing it to anticipate inactivity already before darkness onset. Consequently, an animal's daily behaviour is a mixture of immediate responses to environmental changes and clock-controlled processes. It is not always easy to distinguish between the two contributions, especially not under natural conditions, where multiple environmental factors are mutually changing. Immediate responses to the environment are usually important for fine-tuning clock-controlled responses, but they can be strong enough to conceal the clock-controlled processes, a phenomenon known as ‘masking’ of the endogenous clock output [46].

Transferring animals to the laboratory, and especially to constant conditions, helps to see which parts of activity are clock controlled. This procedure has been carried out for many animals in the last century. Under constant conditions, fruit flies exhibit bimodal activity, with a smaller activity bout in the subjective morning (M, best visible at temperatures above 25°C) and a larger activity bout (E) spanning the subjective afternoon and evening [41,47]. LD cycles in the laboratory modify the shape of M and E activity bouts: they become higher and narrower, couple to lights on and off, respectively, and are clearly separated by a siesta (e.g. [35,41,47]). The phase of these sharp M and E activity peaks in LD can easily be determined. Under LDR1, M and E activity bouts become even sharper and the peaks occur at specific irradiances: approximately 7.5 lux in a previous study [8], and between 1 and 10 lux in this study. The question is: are these sharp peaks the output of an entrained circadian clock or induced by light?

### (a) Sharp M and E peaks are not controlled by the circadian clock

This study clearly shows that sharp M and E peaks provoked by twilight simulation are not the outputs of the circadian clock. Activity of the clock mutants peaked at the same irradiance as activity of WT flies (1–10 lux). Moreover, when irradiance was gradually increased/decreased to/from the same maximal intensity but within 4.5 h instead of 1.5 h, the activity of all flies still peaked at 1–10 lux. Since 1–10 lux was now reached significantly later/earlier, the peaks were shifted, respectively. This behaviour can be fully explained by an immediate response of the flies to the increasing/decreasing irradiance sensed by the compound eyes. Indeed, the sharp peaks completely disappeared in eyeless flies. Obviously, fly activity is stimulated by the increasing/decreasing irradiance, and flies prefer to be active at rather low irradiances. This observation fits with previous results obtained in the laboratory, which have shown that flies show a preference for rather dim light [8] and clock mutants exhibit the same light preference [39].

The perhaps most surprising result of this study is the fact that the activity pattern of *per^01^* and *tim^01^* clock mutants looks virtually identical to that of WT flies when irradiance is slowly increased/decreased, closely mimicking the natural time course of irradiance during the 24 h day. Thus, we could largely reproduce the results of Vanin *et al*. [7], who recorded flies outside the lab under natural-like light and temperature cycles, and found similarly timed onsets of M and E activity in WT flies and *per^01^* and *tim^01^* mutants. Our results also suggest that, in the outdoor experiments of Vanin *et al*. [7], clock mutants may have mainly responded to the cyclic environment, although it is still possible that they possess a residual clock (see the electronic supplementary material). Furthermore, our study indicates that natural-like LD cycles are sufficient to provoke almost normal activity patterns in clock mutants; additional temperature cycles seem not to be necessary.

Nevertheless, outdoor temperature cycles have most likely contributed to the WT-like activity pattern of clock mutants. High temperatures have furthermore caused the so-called ‘afternoon peak’ that appeared in the outdoor experiments during warm days and that was interpreted as an escape response of the flies [5,7]. Menegazzi *et al*. [6] and to some extent also Green *et al*. [48] found the afternoon peak to be more pronounced in clock mutants than in WT flies. This supports the idea that the afternoon peak is a direct response to high temperature and may be partly repressed by the circadian clock of WT flies that usually rest at this time. Here, we found that clock mutants were more active during siesta than WT flies, which fits with this idea. In addition, we show that clock mutants responded more strongly than WT flies to twilight simulations in the middle of the night, a time at which flies normally sleep [47,48]. These findings support our conclusion that a functional clock suppresses activity at unfavourable times. Activity during the hottest part of the day would require a cooling system, whereas activity during the coldest part of the day would need an internal heating system dissipating considerable amounts of energy. Consequently, activity suppression during these times prevents flies from wasting energy. Amazingly, this suppression also works at the constant temperature of 20°C applied here, clearly speaking for an endogenous control.

### (b) The circadian clock responds to twilight

Although the circadian clock is not necessary for the exact timing of M and E peaks under twilight conditions, the molecular clock is receptive to twilight, as we show here for TIM cycling in the clock neurons. TIM accumulated earlier and was present for longer under LDR2 than under LD in all clock neurons. TIM plays a relevant role in clock sensitivity to light because it gets degraded via the proteasomal pathway after interaction with CRY and JETLAG, as soon as the fly perceives light in the morning [16,17,33,51–53]. Similarly, TIM cannot re-accumulate before dusk. In our study, the gradual increase/decrease in irradiance under LDR2 obviously allowed TIM to remain longer in the morning and to increase earlier in the evening. Interestingly, this difference disappeared in *cry^01^* mutants, supporting the idea that CRY is critical for timing the molecular clock in response to twilight. However, the absence of CRY not only impaired sensitivity of the molecular clock to twilight, but also delayed the timing of TIM accumulation and degradation in the 5th s-LN_v_ that belongs to the E neurons (under LD and LDR2). Interestingly, the delay of this E neuron had only minor consequences on the timing of the E activity bout. Only under LD, E activity of *cry^01^* mutants rose slower than in WT flies and the E peak appeared not quite at its maximal level at lights off (figure 2*e*; electronic supplementary material, figure S2A). Furthermore, the *cry^01^* mutants shown in the electronic supplementary material, figure S2A showed more activity after lights off than WT flies and could be stimulated to a higher degree by nocturnal light than WT flies (electronic supplementary material, figure S2B). This indicates that *cry^01^* mutants have a delayed E clock and can therefore be easily activated by nocturnal light. That the degree of light-induced activity depends on the state of the clock has been shown some years ago [40], and that *cry^01^* mutants have a late E activity bout and thus a late E clock was recently demonstrated under long photoperiods [54]. After elimination of the clock in *cry^01^* mutants (*per^01^;;cry^01^* double mutants), nocturnal activity is strongly reduced, as only the responses to twilight are evident. This confirms our conclusion that the high nocturnal activity of *cry^01^* mutants is indeed due to a late E clock. Nevertheless, under LDR1 and LDR2 the behavioural differences between *cry^01^* mutants and WT flies disappeared, suggesting that M and E peak timing by the compound eyes dominated over the effects of CRY on the molecular clock.

### (c) The eyes as important light-sensing organs

Here, we show that light is the key signal for adapting the activity pattern of *Drosophila* to natural-like conditions and that the compound eyes play a major role in sensing gradual changes in light intensity. This makes sense, as the compound eyes mediates other immediate responses of light reported previously, such as the startle response after lights on in LD cycles and the nocturnal increase in activity upon moonlight [39]. To measure gradually changing light intensity, an irradiance-detecting system employing several photopigments is required [53]. Indeed, we showed recently that fruit flies use multiple photopigments within their compound eyes to detect dim light [22] and twilight [23].

## 5. Conclusion

In summary, our study is encouraging for organisms carrying clock gene mutations. Obviously, nature provides mechanisms that allow for almost normal activity rhythms. The natural LD cycle and functional eyes seem to be sufficient for normal timing. In nature, temperature cycles and other cycling Zeitgebers can contribute to fine-tune activity, making it difficult to distinguish clock mutants from animals with normally ticking clocks.

## Supplementary Material

Supplementary Material

## Data accessibility

Additional discussion, two tables and three figures are uploaded as electronic supplementary material. Original data are available at http://dx.doi.org/10.5061/dryad.90kj2.
