# Supplementary material for: Normal vision can compensate for the loss of the circadian clock
Source: Proc Biol Sci. 2015 Sep 22;282(1815):20151846. doi: 10.1098/rspb.2015.1846 (PMC4614763; doi:10.1098/rspb.2015.1846)
Supplement: Supplementary Material [file rspb20151846supp1.pdf]

SUPPLEMENTARY MATERIAL

Supplementary Discussion

Does the lack of anticipatory morning activity in our study mean that the M oscillator is not endogenous but rather a response to light?

In contrast to other studies (e.g. [1,2]), the wild-type flies of our present study show very little if any anticipatory activity of lights-on under rectangular LD-cycles (Fig. 1, Fig. S1). It even appears that the clock mutants, especially per^0 mutants, show more anticipatory M activity than the WT flies (Fig. 1). Most significantly, under outdoor conditions, M activity is also not anticipatory but seems to be provoked by dawn [3]. Furthermore, M activity strongly depends on environmental temperature and is suppressed under cold days [3]. All this suggests that M activity is not controlled by the endogenous clock but is instead a response to lights-on/twilight under favorable temperatures. However, this appears rather unlikely, because (1) M and E activity bouts can also be observed under temperature cycles alone [4], (2) both activity bouts persist after transferring the flies into constant conditions, especially at high environmental temperatures [5] and (3) in rare cases M activity is free-running with a different period than E activity [6]. All this demonstrates that M activity is of endogenous nature and even independently controlled from E activity as also suggested by the popular M and E oscillator model [7-9]. Thus, there must be other reasons for the lack of M anticipation under outdoor conditions and the present experiments. One obvious reason is temperature. Our experiments are performed at 20°C whereas other labs use 25°C as recording temperature. The second reason for the lack of M anticipation is the recording system [10]. In the present experiments, we used our home-made recording system in which flies are placed in photometer cuvettes with an infrared-light beam on the opposite side of the cuvette where water and sugar is offered. In order to cross the infrared light beam, the flies would have to cover the whole distance of the cuvette, which almost never happens during the night or in the early morning based on analyzing the running profile of flies using a camera based

system [10]. During dark nights, flies appear to cover only smaller distances, which appears reasonable since they cannot see anything. In the commercial Trikinetics system used by most laboratories, even such small movements are registered, which might be explained by the small tube volume that allows IR beam crossings with little activity. Indeed, even at 20°C, flies recorded by the Trikinetics system show anticipatory M activity [10].

We conclude that M anticipatory activity depends on environmental temperature as well as on the recording system and is not an adequate criterion for clock-controlled activity (see also discussion in [10]).

Do *per^{01}* and *tim^{01}* mutants have a residual clock that facilitates wild-type activity patterns under more natural-like conditions?

The next question to be answered is why the clock mutants *per^{01}* and *tim^{01}* have even more anticipation of lights-on than WT flies (Fig. 1). If the ability to anticipate lights-on is taken as proof for an endogenous clock, the mutants must at least have a working M oscillator. Indeed, there are old observations that point to a residual M oscillator in *per^{01}* mutants [11-12]. According to these findings the M oscillator of *per^{01}* mutants is not strong enough to drive locomotor activity rhythms under constant conditions but it is strong enough to promote activity before lights-on in the morning; moreover, M-activity-onset depends on the Zeitgeber period being earlier under long periods [12]. WT flies show the same dependency of M-activity-onset on Zeitgeber period, but they always begin activity ~2h after *per^{01}* mutants. This result fits nicely to the here observed stronger anticipation of lights-on in *per^{01}* mutants. Several other observations point to the presence of a residual clock in *per^{01}* and *tim^{01}* mutants: individual mutant flies showed bimodal activity with a real siesta and other individuals exhibited even rhythmic activity for some days under constant conditions [4, 12-15]. In the present study we could also see bimodal activity in some *tim^{01}* mutants under LD and this is reflected in the average activity profile shown in Figure 2A. *per^{01};cry^{b}* double mutants have even been reported to show more robust bimodal activity patterns under LD conditions than *per^{01}* single mutants, perhaps because the absence of functional CRY facilitates photoreceptor input from the

compound eyes, thereby provoking a more clear-cut synchronization of the residual oscillator [14].

The molecular basis of the residual oscillator is not completely clear. It is possible that the *per* and *tim* genes, respectively, have some residual function in the absence of one of them (see [14]). However, even $per^{01};tim^{01}$ mutants show some residual rhythmicity [4], suggesting that the second molecular feed-back loop comprising *vrille* and *Pdp1* (reviewed in [16]) can still show some rhythmic activity in absence of *per* and *tim*. The *Clk* gene is central to both feed-back loops [16]. Therefore, Clk^{JRK} mutants should not show any rhythmicity. This is indeed the case [4]. Thus, it is well conceivable that the residual clocks of per^{01} and tim^{01} mutants may have contributed to their quasi-normal activity under LDR2 and outdoor conditions [3]. Most importantly, twilight simulations considerably improve synchronization of several species to light cycles [16-25]. Not only the precision of the synchronized rhythm is higher, but also re-entrainment to shifted LD-cycles occurs faster and the animals can adapt better to unusually long photoperiods and Zeitgeber cycles with different periods. Twilight may therefore have improved rhythmic output in animals with a weak residual clock such as per^{01} and tim^{01} mutants.

Literature

1. Hamblen-Coyle MJ, Wheeler DA, Rutila JE, Rosbash M, Hall JC. 1992 Behavior of period-altered rhythm mutants of *Drosophila* in light-dark cycles. *J. Insect Behav.* **5**, 417-446.
2. Wheeler DA, Hamblen-Coyle MJ, Dushay MS, Hall JC. 1993 Behavior in light-dark cycles of *Drosophila* mutants that are arrhythmic, blind, or both. *J. Biol. Rhythms* **8**(1), 67-94.
3. Vanin S, Bhutani S, Montelli S, Menegazzi P, Green EW, Pegoraro M, Sandrelli F, Costa R, Kyriacou CP. 2012 Unexpected features of *Drosophila* circadian behavioural rhythms under natural conditions. *Nature 484*, 371-375. (doi:10.1038/nature10991).

4. Bywalez W, Menegazzi P, Rieger D, Schmid B, Helfrich-Förster C, Yoshii T (2012) The dual oscillator system of *Drosophila melanogaster* under natural-like temperature cycles. *Chronobiol. Int.* **29**, 395-407. (doi: 10.3109/07420528.2012.668505).

5. Majercak J, Sidote D, Hardin PE, Edery I. 1999. How a circadian clock adapts to seasonal decreases in temperature and day length. *Neuron* **24**(1), 219-230.

6. Helfrich-Förster C. 2000 Differential control of morning and evening components in the activity rhythm of *Drosophila melanogaster* - sex specific differences suggest a different quality of activity. *J. Biol. Rhythms* **15**, 135-154.

7. Grima B, Chélot E, Xia R, Rouyer F. 2004 Morning and evening peaks of activity rely on different clock neurons of the *Drosophila* brain. *Nature* **431**(7010), 869-873.

8. Stoleru D, Peng Y, Agosto J, Rosbash M. 2004 Coupled oscillators control morning and evening locomotor behaviour of *Drosophila*. *Nature* **431**(7010), 862-868.

9. Rieger D, Shafer OT, Tomioka K, Helfrich-Förster C (2006) Functional analysis of circadian pacemaker neurons in *Drosophila melanogaster*, *J. Neurosci.* **26**(9), 2531–2543.

10. Schlichting M, Helfrich-Förster C. 2015 Photic Entrainment in Drosophila assessed by Locomotor Activity Recordings. In *Methods in Enzymology: Circadian Rhythms and Biological Clocks* (ed. Sehgal A.), pp. 387-405, Elsevier.

11. Helfrich C, Engelmann W. 1987. Evidences for circadian rhythmicity in the per^0 mutant of *Drosophila melanogaster*. *Z. Naturforsch.* **42**, 1335-1338.

12. Helfrich-Förster C. 2001 The activity rhythm of *Drosophila melanogaster* is controlled by a dual oscillator system. *J. Insect Physiol.* **47**, 877-887.

13. Kempinger L, Dittmann R, Rieger D, Helfrich-Förster C. 2009 The nocturnal activity of fruit flies exposed to artificial moonlight is partly caused by direct light effects on the activity level that bypass the endogenous clock. *Chronobiol. Int.* ***26***, 151-166.

14. Collins BH, Dissel S, Gaten E, Rosato E, Kyriacou CP. 2005. Disruption of Cryptochrome partially restores circadian rhythmicity to the arrhythmic period mutant of *Drosophila*. *Proc. Natl. Acad. Sci. U. S. A.* **102**, 19021-19026.

15. Yoshii T, Sakamoto M Tomioka K. 2002 A temperature-dependent timing mechanism is involved in the circadian system that drives locomotor rhythms in the fruit fly *Drosophila melanogaster*. *Zool. Sci.* **19**, 841-850.
16. Hardin PE. 2011 Molecular genetic analysis of circadian timekeeping in *Drosophila*. *Adv. Genet.* **74**, 141-173.
17. Boulos Z, Macchi M, Terman M. 1996 Effects of twilights on circadian entrainment patterns and reentrainment rates in squirrel monkeys. *J. Comp. Physiol.* **179**, 687-694.
18. Boulos Z, Macchi M Terman M. 1996 Twilight transitions promote circadian entrainment to lengthening light-dark cycles. *Am. J. Physiol.* **271**, R813-818.
19. Boulos Z Macchi MM. 2005 Season- and latitude-dependent effects of simulated twilights on circadian entrainment. *J. Biol. Rhythms* **20**, 132-144.
20. Boulos Z, Macchi MM, Terman M. 2002 Twilights widen the range of photic entrainment in hamsters. *J. Biol. Rhythms* **17**, 353-363.
21. Comas M, Hut RA. 2009 Twilight and photoperiod affect behavioral entrainment in the house mouse (*Mus musculus*). *J. Biol. Rhythms* **24**, 403-412.
22. Danilenko KV, Wirz-Justice A, Krauchi K, Weber JM, Terman M. 2000 The human circadian pacemaker can see by the dawn's early light. *J. Biol. Rhythms* **15**, 437-446.
23. Gorman AM, Zucker I. 1998 Mammalian seasonal rhythms: new perspectives gained from the use of simulated natural photoperiodes. In Biological Clocks: Mechanisms and Applications, Y. Touitou, ed. (Amsterdam: Elsevier), pp. 195-204.
24. Kavaliers M, Ross DM. 1981 Twilight and day length affect the seasonality of entrainment and endogenous circadian rhythms in a fish, *Couesius plumbeus*. *Can. J. Zool.* **59**, 1326-1334.
25. Kavaliers M, Hirst M, Teskey GC. 1984 Aging and daily rhythms of analgesia in mice: effects of natural illumination and twilight. *Neurobiol. Aging* **5**, 111-114.

Supplementary Tables

Table S1

Statistics (Two-Way ANOVA) for E peak timing and nocturnal activity in $WT_{CantonS}$ and clock mutants (*per⁰*, *tim⁰*), and photoreceptor mutants with (*cli^eya*, *cry^01*) or without (per⁰;*cli^eya*, *per⁰;;cry^01*) a circadian clock

	**$WT_{CantonS}$ and clock mutants (*per^0*, *tim^0*)**	
	E peak timing	Nocturnal activity
Dependence on light condition (LDR1/LDR2)	$F_{(2,164)}$=260.37; p<0.001	$F_{(2,211)}$=85.26; p<0.001
Dependence on genotype	$F_{(2,164)}$=24.08; p<0.001	$F_{(2,211)}$=20.13; p<0.001
	**Photoreceptor mutants with (*cli^{eya}*, *cry^{01}*) or without (per^0;*cli^{eya}*, *per^0;;cry^{01}*) a circadian clock**	
	E peak timing	Nocturnal activity
Dependence on light condition (LDR1/LDR2)	$F_{(2,231)}$=53.49; p<0.001	$F_{(2,231)}$=43.11; p<0.001
Dependence on genotype	$F_{(2,231)}$=333,83; p<0.001	$F_{(1,860)}$=8.22; p<0.001

Table S2

Statistics (Three-Way ANOVA) for TIM increase/decrease in the clock neurons of $WT_{CantonS}$, $WT_{Lindelbach}$ and cry^{01} mutants

	**TIM increase**		
	$WT_{CantonS}$ (ZT10-16)	$WT_{Lindelbach}$ (ZT12-18)	cry^{01} (ZT10-16)
Dependence on time	$F_{(3,447)}$=932.27; p<0.001	$F_{(3,237)}$=243.53; p<0.001	$F_{(3,447)}$=110.76; p<0.001
Dependence on light	$F_{(1,447)}$=221.96; p<0.001	$F_{(1,237)}$=87.44; p<0.001	$F_{(1,447)}$= 2.06; p=0.152
Dependence on clock neuron group	$F_{(5,447)}$=105.30; p<0.001	$F_{(5,237)}$=18.97; p<0.001	$F_{(5,447)}$=120.81 p<0.001
Interaction between time and light	$F_{(3,447)}$=29.90; p<0.001	$F_{(3,237)}$=18.48; p<0.001	$F_{(3,447)}$=1.08; p=0.358
Interaction between neuron and light	$F_{(5,447)}$=4.94; p<0.001	$F_{(5,237)}$=0.86; p=0.508	$F_{(5,447)}$=0.19; p=0.966
Interaction between time and neuron	$F_{(15,447)}$=10.46; p<0.001	$F_{(15,237)}$=1.81; p=0.035	$F_{(15,447)}$=10.18; p<0.001
	**TIM decrease**		
	$WT_{CantonS}$ (ZT22-2)	$WT_{Lindelbach}$ (ZT22-2) [a)]	cry^{01} (ZT22-2)
Dependence on time	$F_{(1,447)}$=837.78; p<0.001	$F_{(3,860)}$=502.65; p<0.001	$F_{(3,447)}$=204.70; p<0.001
Dependence on light	$F_{(1,447)}$=59.34; p<0.001	$F_{(1,860)}$=120.23; p<0.001	$F_{(1,447)}$=0.04; p=0.834
Dependence on clock neuron group	$F_{(5,447)}$=56.29; p<0.001	$F_{(5,860)}$=66.11; p<0.001	$F_{(5,447)}$=73.82; p<0.001
Interaction between time and light	$F_{(3,447)}$=53.67; p<0.001	$F_{(3,860)}$=34.95; p<0.001	$F_{(3,447)}$=2.31; p=0.076
Interaction between neuron and light	$F_{(5,447)}$=14.74; p<0.001	$F_{(5,860)}$=22.16; p<0.001	$F_{(5,447)}$=1.63; p<0.151
Interaction between time and neuron	$F_{(15,447)}$=3.72; p<0.001	$F_{(20,860)}$=4.70; p<0.001	$F_{(15,447)}$=2.64; p<0.001

[a)]TIM decrease in $WT_{Lindelbach}$ was assessed every hour. There are hence more time-points and consequently a higher sample size in this column.

Supplementary Figures

Figure S1

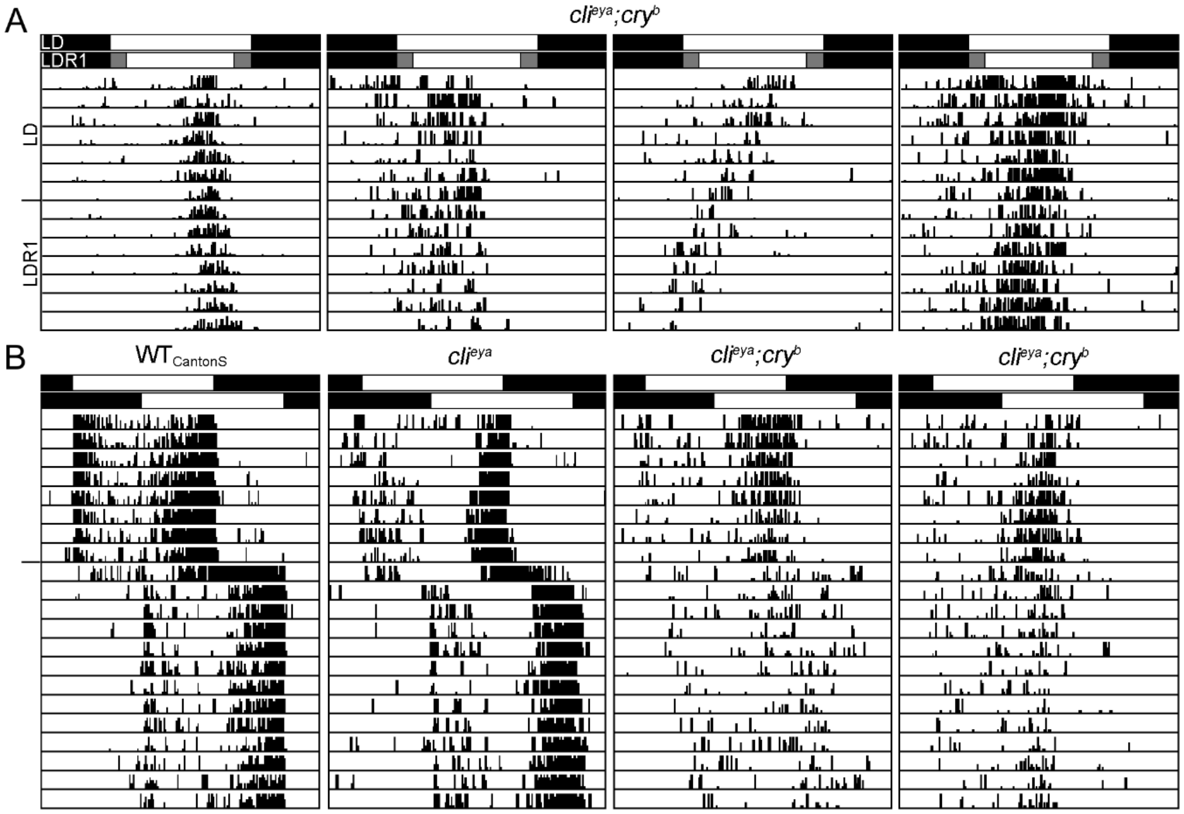

<u>Figure S1:</u> Mutants lacking the compound eyes plus functional cryptochrome (*cli*^{eya}*;cry*^{b}) hardly entrain to LD and LDR cycles. A: Actograms of four individual *cli*^{eya}*;cry*^{b} double mutants. All flies neither entrained to LD nor to LDR1, but instead free-run with a period close to 24h. Please note that the free-run of the individual flies roughly starts from the same phase, indicating that the flies have been entrained during rearing. Nevertheless, the here chosen LD-cycles (100 lux light-intensity during the day) are not strong enough to provoke clear entrainment. **B:** Actograms of a WT fly, a *cli*^{eya} mutant and two *cli*^{eya}*;cry*^{b} double mutants that were subjected to a 6h phase delay of the LD cycle on day 9 of recording. Whereas the WT fly phase-delays activity within 1 day, the *cli*^{eya} mutant needs approximately 2 days. The *cli*^{eya}*;cry*^{b} double mutants do not phase-shift at all.

Figure S2

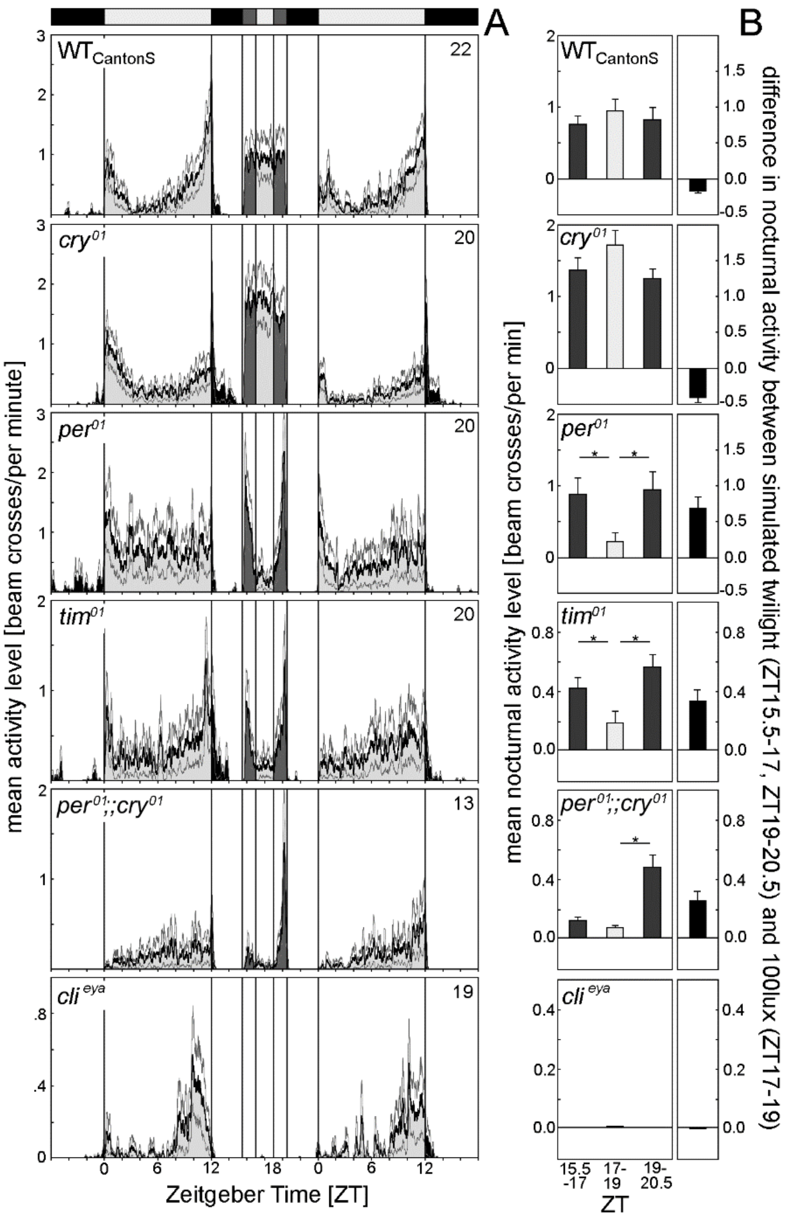

<u>Figure S2:</u> Simulated twilight in the middle of the night can stimulate clock-less mutants to high activity. Flies were entrained in LD12:12 for 5 days. On day 6 we applied a light pulse in the middle of the night with dawn from ZT15.5-17 and dusk from ZT19-20.5. **A:** The activity profiles represent the mean average activity profiles of days 6 and 7 (black line) ± SEM (gray lines). Times of total darkness are depicted in black, dawn/dusk simulation in dark gray and

times of highest light intensity (100 lux) in light gray. Most genotypes respond to the "midnight pulse" by increasing activity, only eyeless flies do not respond at all. Clock mutants (*per*^{01}, *tim*^{01} and *per*^{01}*;;cry*^{01}) show a bimodal pattern during dawn and dusk simulation in the middle of the night, whereas WT and *cry*^{01} flies only show an increase of activity without sharp peaks. This indicates that the sharp M and E peaks in LDR1/LDR2 are direct effects of light caused by the compound eyes. **B:** Mean activity levels during simulated midnight dawn/ midnight dusk and the two hours of 100 lux in between. WT flies and *cry*^{01} mutants show the same amount of activity during all three conditions, whereas the clock-less mutants have a significantly higher activity level during the times of simulated twilight. Asterisks ($p < 0.05$) mark significant differences. The right panels show the differences in mean activity level between the times of simulated twilight (means of dawn and dusk simulation) and the two hours of 100 lux. For details see text.

Figure S3

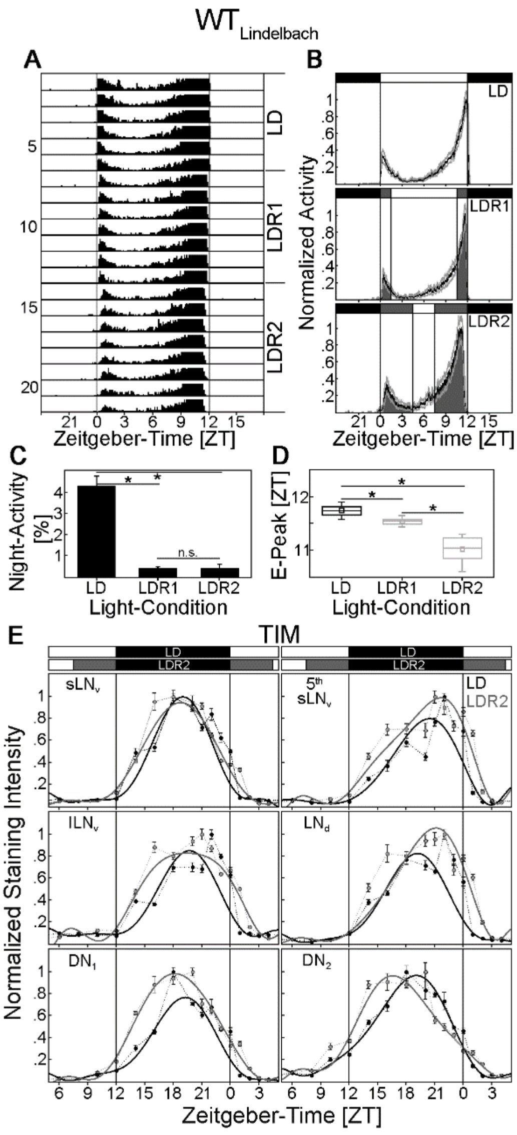

Figure S3: Entrainment of WT$_{\text{Lindelbach}}$ flies to LD, LDR1 and LDR2 and TIM cycling in LD and LDR2. For locomotor activity an average actogram (A), average activity profiles (B), nocturnal activity (C) and timing of E activity peaks (D) were calculated. In the average actogram (A), the mean activity of 25 flies is indicated in black without error bars. LD was simulated from day 1-6, LDR1 from day 7-13 and LDR2 from day 14-21. The gray vertical lines indicate Zeitgeber Time (ZT) 0 (= beginning of day) and ZT 12 (= beginning of night). The average activity profiles (B) indicate the average activity of all flies (black curve) ±SEM (light gray) under the relevant light condition (LD, LDR1, LDR2), which is given on top of each

diagram (black: complete darkness, dark gray: time of increasing or decreasing light intensity, white: time of maximal light intensity (100 lux)). The flies show bimodal activity patterns with M and E activity bouts under all three light conditions. Upon LDR1, and more so upon LDR2, nocturnal activity decreases (C) and E peaks advance (D). Nocturnal activity is expressed as a percentage of whole daily activity (± SEM), timing of E peak in ZT. **E:** TIM cycling is investigated in LD and LDR2 in the following 6 different neuron cluster: sLN_v, 5th sLN_v, lLN_v, LN_d, DN_1 and DN_2. Black circles (± SEM) connected by thin broken black lines represent the measured staining intensity in LD, whereas gray circles (± SEM) connected by thin gray broken lines represent the staining intensity in LDR2. Polynomial fits of the cycling in LD and LDR2 are added in thick black and gray lines, respectively. The polynomial fits are characterized by $R^2 \geq 0.98$ indicating that they nicely match the original cycling. TIM accumulates earlier and stays stable for longer time in LDR2 as compared to LD in all neurons. The calculation of the kurtosis of TIM distribution under LDR2 and LD confirms this observation: kurtosis is more negative under LDR2 than under LD, meaning that TIM distribution is broader under LDR2. ANOVA confirms that kurtosis of TIM distribution is significantly dependent on the light-condition ($F_{(1,10)}=8.199$; $p=0.017$). In addition, TIM peaks earlier in the DN_2 under LDR2 ($F_{(1,38)}=9.069$; $p=0.015$).
